# An Original and Efficient Antibiotic Adjuvant Strategy to Enhance the Activity of Macrolide Antibiotics against Gram-Negative Resistant Strains

**DOI:** 10.3390/ijms232012457

**Published:** 2022-10-18

**Authors:** Azza Troudi, Jean Michel Bolla, Naouel Klibi, Jean Michel Brunel

**Affiliations:** 1 Aix Marseille Université, INSERM, SSA, MCT, 13385 Marseille, France; 2 Laboratory of Microorganisms and Active Biomolecules, Department of Biology, Faculty of Sciences of Tunis, University of Tunis El Manar, Tunis 1068, Tunisia

**Keywords:** polyaminoisoprenyl derivatives, antibiotic adjuvants, polyamines, Gram-negative bacterial strains, macrolide antibiotics, outer membrane

## Abstract

Gram-negative bacteria were reported as a significant cause of infections in both community and nosocomial settings. Considered as one of the greatest threats to public health, the spread of bacteria drug resistance and the lack of effective alternative treatment options remains problematic. Herein, we report a promising strategy to combat Gram-negative resistant strains consisting of the combination of a macrolide antibiotic with a polyaminoisoprenyl adjuvant derivative leading to a significant decrease of antibiotic resistance.

## 1. Introduction

The discovery of beta-lactam antibiotics in 1928 by Fleming was revolutionary and has saved countless lives from severe infectious diseases caused by bacterial strains [1,2,3]. Nevertheless, the excessive use of these drugs in many fields of medicine has contributed to the emergence of bacterial resistance and leading to limited treatment options [4,5]. The fast rate of spread of multidrug-resistant Gram-negative bacteria remains an important concern due to their intrinsic resistance and their ability to rapidly develop new mechanisms of resistance [6,7]. In this context, *Enterobacteriaceae*, such as *Klebsiella pneumoniae* and *Escherichia coli*, as well as *Pseudomonas aeruginosa* and *Acinetobacter* spp., have been identified as responsible for most multidrug-resistant bacterial infections [8,9,10]. However, antibiotic resistance represents a natural phenomenon that cannot be stopped and innovative approaches to restore antibiotic failure are desperately needed. In this context, it has been previously demonstrated that combined antibiotic therapy using macrolide antibiotics such as erythromycin, clarithromycin, and azithromycin could present high anti-biofilm activity both in vitro and in vivo [11]. Another approach currently under investigation is the design and synthesis of new classes of adjuvants able to restore the activity of inefficient antibiotics [12].

Gram-negative bacteria are intrinsically resistant to many compounds due to their outer membrane (OM) composition. Thus, it is well known that their cell envelope is a complex multilayered structure where bacteria are surrounded by a thin peptidoglycan cell wall, which itself is surrounded by an outer membrane containing lipopolysaccharide (LPS). In the complex OM architecture lipid A, the hydrophobic group of lipopolysaccharide covers the surface of most Gram-negative bacteria, playing an essential role by anchoring the lipopolysaccharide in the membrane [13]. Furthermore, the spatial organization of LPS molecules is stabilized by Mg^2+^ and Ca^2+^ divalent cations, resulting in a barrier that is difficult to penetrate by numerous classes of antibiotics [14,15,16,17,18,19]. Thus, this OM obstacle needs to be circumvented since it has proven to be especially problematic to modern target-based antibacterial drug design. OM perturbation can be achieved in vitro through a variety of genetic means [17,20,21]. However, the effects of such genetic perturbation are mostly permanent [22,23]. On the other hand, well-known compounds such as EDTA increase the permeability of the OM by chelating the divalent cations stabilizing LPS [24,25]. Other chemical compounds, most notably polymyxin derivatives, can bind the lipid A displacing the divalent cations and, thus, disrupting the OM integrity [26,27,28,29,30]. Nevertheless, despite being good OM permeabilizers, their inner membrane (IM) activity makes these compounds mainly nonspecific [31].

For more than six decades, the clinical use of macrolides increased gradually since their discovery [32]. Known for their mechanism of action of inhibiting protein synthesis by targeting the bacterial 50S ribosomal subunit, almost all these drugs present similar antibacterial profiles and are mainly active against Gram-positive bacteria [33]. This family of antimicrobials has also been used against specific and limited species of Gram-negative bacteria such as azithromycin, commonly used against *Enterobacteriaceae* infections [34].

To discover new compounds capable of perturbing the Gram-negative OM, our group has recently developed the design and use of polyaminoisoprenyl derivatives as antibiotic enhancers exhibiting a strong effect on the level of tetracycline antibiotics susceptibility against resistant *P. aeruginosa* bacterial strains [35,36,37,38]. In the continuing course of our studies, we report herein a promising strategy to combat Gram-negative resistant strains consisting of the combination of a macrolide antibiotic with polyaminoisoprenyl adjuvant derivatives leading to a significant decrease of antibiotic resistance and increase of Gram-negative strains susceptibility towards macrolides. We have also investigated the efficiency of such an approach against the most common bacterial strains and established a close resistance profile–activity relationship.

## 2. Results

### 2.1. Synthesis of Polyaminoisoprenyl Derivatives ***3**–**6***

The synthesis of polyaminoisoprenyl derivatives **3**–**6** utilized an optimized direct nucleophilic substitution of the appropriate polyamine on farnesyl chloride **1** and neryl chloride **2** performed in THF at room temperature for 12 h (Table 1). Performing this reaction for 24 h led to the formation of a higher proportion of by-products. Under the 12 h reaction conditions, the expected products were obtained as pure isomers in yields ranging from 49 to 72%, respectively. Cytotoxicity was evaluated against Chinese hamster ovary (CHO) cells, with all compounds presenting IC_50_ ranging from 126 to 150 µM, suggesting that they were minimally toxic. 

### 2.2. Antimicrobial Activity of Polyaminoisoprenyl Derivatives ***3**–**6*** against Gram-Negative Bacteria

Table 2 summarizes the MICs obtained for the polyaminoisoprenyl derivatives **3**–**6** against Gram-negative strains. Compounds **3**–**6** demonstrated a similar behavior with MICs ranging from 50 to greater than 200 µM, whereas compound **3** presented MICs from 12.5 to 100 µM depending on the considered bacterial strains.

### 2.3. MICs of the Different Macrolides Tested against Various Gram-Negative Bacteria

**Figure 1:**
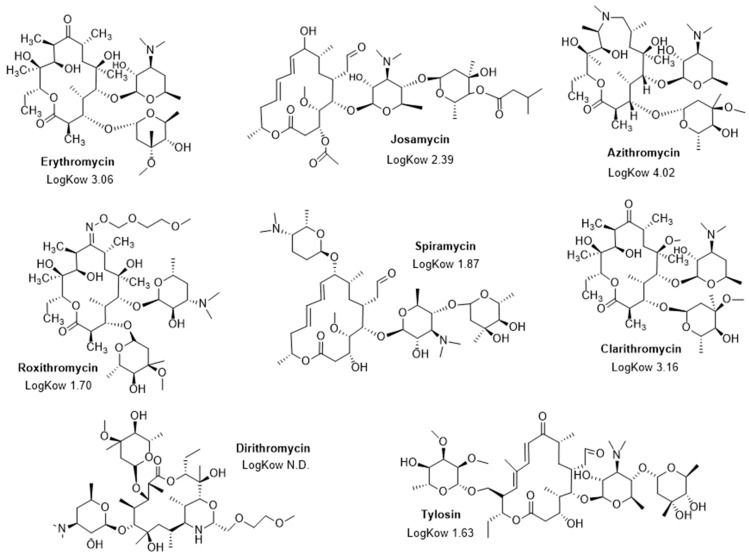
Structure of the macrolides used in this study and their associated LogKow values.

The MICs of different macrolides (erythromycin, josamycin, roxithromycin, azithromycin, spiramycin, clarithromycin, dirithromycin, and tylosin) (Figure 1) were determined against all the Gram-negative bacterial strains, with MIC’s ranging from 2 µg/mL to greater than 1024 µg/mL (Table 3). In this context azithromycin appeared as the most effective antibiotic against all the selected bacteria with MICs varying from 2 to 128 µg/mL, whereas all the other tested macrolides led to higher MICs ranging from 128 to 1024 µg/mL.

### 2.4. Restoration of Macrolides Activity against Various Gram-Negative Bacteria in Combination with Derivatives ***3**–**6***

MICs of the macrolides in combination with the polyaminoisoprenyl derivatives were determined to evaluate the antibiotic enhancing activities of **3**–**6** toward numerous Gram-negative bacterial strains (Table 4). Compound **3** used at a 10 µM concentration increased the susceptibility of all the bacterial strains with respect to all the tested antibiotics by improving their antimicrobial activities. It is noteworthy that compound **4** led to some enhancement but in a less efficient manner than compound **3**. Interestingly, under the same experimental conditions, the parent geranyl derivatives **5** and **6** demonstrated only weak ability to restore the activity of the macrolides toward Gram-negative bacteria. 

Considering macrolides as OM-impermeable antibiotics, their antibacterial spectrum is restricted mostly to Gram-positive organisms [39]. Potentiation of these antibiotics by compound **3** was prevalent against all wild-type (except AG100A puc) Gram-negative bacteria tested. The mechanism of action of macrolides is well known to involve inhibition of bacterial protein synthesis, and they were rendered active against Gram-negative bacteria by the adjuvant allowing them to overcome the OM barrier. Since compound **3** (with a farnesyl and a long polyamine chain (spermine)) is more potent than compound **4** (with a ramified polyamine group) and derivatives **5** and **6** (with a shorter geranyl moiety), it clearly appears that both the presence of a long hydrophobic carbon chain and a highly charged linear polyamine group bestow preferential membrane interactions relative on these compounds. All these assumptions tend to suggest that these compounds can disrupt the OM integrity facilitating the entrance of hydrophobic antibiotics. It is also noteworthy that the ability of compound **3** to enhance macrolide’s activity was further evaluated against MDR clinical isolate Ea289 with similar success than toward sensitive ones.

The value of the Fractional Inhibitory Concentration (FIC index) as a predictor of synergy was investigated using the various macrolides as antibacterial agents combined with compound **3** in fully blind experiments against numerous different bacterial strains (Table 5). Under the conditions used, except for PA01 strain for which no synergy was observed whatever the considered macrolide, synergies were encountered with FIC index <0.25 in numerous cases, demonstrating the strong effect of compound **3** to restore the antibacterial activity of macrolides against Gram-negative bacteria.

**Figure 2:**
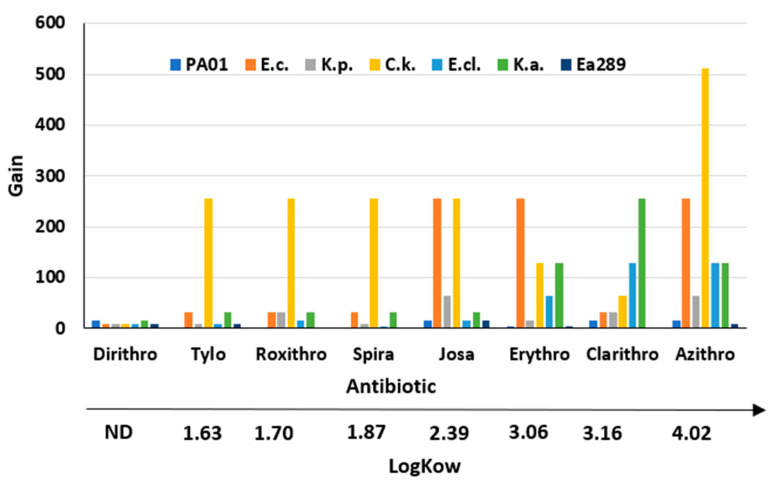
Correlation of the gain factor observed toward numerous Gram-negative bacteria depending on the LogKow value of the considered macrolide antibiotic.

It is interesting to note that some macrolides, such as josamycin, erythromycin, clarithromycin, and azithromycin, are better than others—e.g., dirithromycine, tylosine, roxithromycine, and spiramycine—when combined with compound **3** against the considered bacterial strains. Thus, by considering the gain obtained (as the ratio of the MIC of the macrolide alone to the MIC of the macrolide combined with **3** used at a 10 µM concentration), we clearly notice a strong correlation with the LogKow (values found in PubChem for all the tested macrolides) of the macrolides, commonly used as a measure of hydrophobicity, since, overall, the higher the lipophilicity of the compound, the higher the obtained gain (Figure 1 and Figure 2).

### 2.5. Mechanism of Action of Compound ***3***

#### Inner Membrane Depolarization Assay

The most efficient adjuvant (Cpd **3**) was evaluated for its potent ability to disrupt the proton gradient of the bacterial inner membranes of the Gram-negative bacteria [40]. Thus, to monitor the phenomenon, DiSC_3_(5) assay was used to measure the electrical potential gradient across the inner membrane. DiSC_3_(5) is a cationic membrane permeable fluorescent dye which can build up on hyperpolarized membrane and translocate into the lipid bilayer. If compound **3** interferes with the inner membrane, then it will lead to a membrane depolarization with the dye being consequently released into the external environment. The increase of fluorescence is then recorded and subsequently quantified.

**Figure 3:**
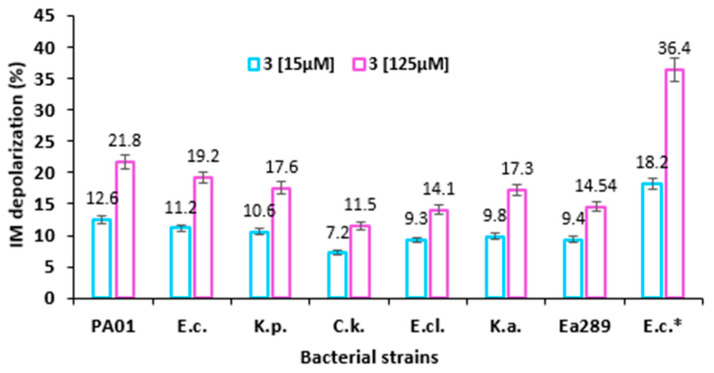
Inner membrane depolarization of various Gram-negative bacteria (PA01, *E. coli* ATCC 25922, *K. pneumoniae* ATCC 13883, *C. koseri* IP8294, *E. cloacae* DSM 129, *K. aerogenes* ATCC 13048, *K. aerogenes* 289 (MDR), and *E. coli* AG100A_pUC18) by evaluating DiSC_3_(5) fluorescence recorded after 5 minutes in the presence of compound **3** at 15 µM and 125 µM. * Refers to *E. coli* AG100A_pUC18. The results represent the average plus SD of three independent experiments.

Figure 3 shows the dose-dependent increase (at two different concentrations, 15 and 125 µM) in the percentages of depolarization (calculated on the basis of untreated controls) of the inner membrane resulting from its alteration by compound **3**. Interestingly, no significant differences were observed between all the considered Gram-negative strains whatever the concentration tested, except for AG100A_pUC18 which does not present any AcrAB efflux pumps expression. In the latter case, the obtained values were almost twofold higher compared with the rest of the strains tested at the same concentration of compound **3**. The other tested concentrations of compound **3** on the inner membrane depolarization of various Gram-negative bacteria are presented in Figure S2.

### 2.6. ATP Efflux Measurement

**Figure 4:**
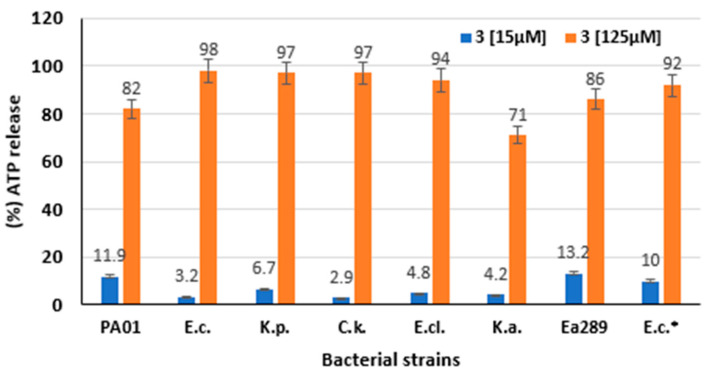
ATP release levels measurement in the presence of compound **3**. ATP release levels of various Gram-negative bacteria (PA01, *E. coli* ATCC 25922, *K. pneumoniae* ATCC 13883, *C. koseri* IP8294, *E. cloacae* DSM 129, *K. aerogenes* ATCC 13048, *K. aerogenes* 289 (MDR), and *E. coli* AG100A_pUC18) evaluated after 1 minute by bioluminescence in the presence of compound **3** at 15 µM and 125 µM. In each case, polymyxin B (250 µM) was used as a positive control to quantify the maximum level of ATP efflux. * Refers to *E. coli* AG100A_pUC18.

A bioluminescence method was utilized to determine the behavior of compound **3** on the intracellular pool of bacterial ATP. Thus, the external concentration of ATP was used as a reporter reflecting the permeabilizing effect of **3** (Figure 4) along with providing a dose–response curve (Figure S3) [41,42]. Thus, **3** used at a 125 µM concentration dose dramatically disrupted the Gram-negative bacterial membranes after 1 min as observed by intracellular ATP release kinetics, which was similar to that observed for the positive control polymyxin B (Figure 4). Conversely, no significant effect was found by using spermine as a polyamine negative control during the test time). Lower but significant ATP efflux was observed after 1 min for compound **3** used at a 15 µM concentration with 2.9 to 13.2% ATP efflux release relative to the CTAB positive control depending on the nature of the considered bacteria, respectively. As illustrated in Figure 4, compound **3** led to a significant level of ATP release against the multidrug-resistant Ea289 strain even at low concentrations, since the percentage of ATP detected was up to twofold higher compared with that of the other strains (see Figure S3 for dose-dependent data).

### 2.7. Outer Membrane Permeabilization

The nitrocefin hydrolysis method was used to evaluate the effect of **3** on the integrity of the outer membranes of the different Gram-negative bacteria. This assay relies upon the hydrolysis of the chromogenic β-lactam nitrocefin periplasmic beta-lactamases, leading to a change in color from yellow to red, relating color change to the degree of integrity of the outer membrane.

**Figure 5:**
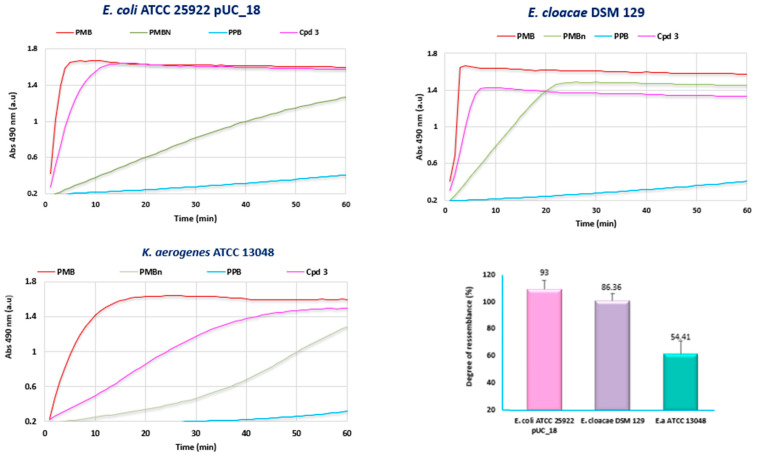
Outer membrane permeabilization of chosen Gram-negative bacterial strains by evaluating the rate of nitrocefin hydrolysis in the presence of PPB (Potassium Phosphate Buffer), PMB, PMBn, and compound **3** at 125 µM.

In this context, Figure 5 presents the results of our investigations against some selected Gram-negative bacteria (producing β-lactamases) (*E. coli* ATCC25922 pUC_18, *E. cloacae* DSM 129, and *K. aerogenes* ATCC 13048) in the presence and absence of compound **3** or two positive controls (Polymyxin B (PmB) and Polymyxin Nona PmNona). 

At a high concentration of 125 µM, compound **3** showed a strong effect on the OM permeability compared with polymyxin B. Interestingly, the increase of the nitrocefin hydrolysis rate in the presence of **3** was highly dependent on the considered Gram-negative bacteria. This rate ranged from 54% to 93% compared with polymyxin B suggesting that the behavior of **3** with respect to the binding of the outer membrane was not similar. The results of the nitrocefin test performed against all the tested Gram-negative strains in this study are presented in Supplementary Data (Figure S4).

## 3. Discussion

Infectious diseases caused by Gram-negative bacterial resistant strains represent a major health concern. Studies have demonstrated that in most multidrug-resistant strains, the alteration of the cell envelope permeability is frequently reported. In previous works, we demonstrated that the OM could play a major role in the susceptibility of these bacteria to antibiotics. Gram-negative bacteria can proceed to the modification of lipid A or proteins in OM composition leading to the resistance or antibiotic susceptibility of the considered species [13]. In a previous study, we determined that the combination of a farnesyl spermine compound **3** used at concentrations ranging from 2.5 to 10 µM, in the presence of doxycycline or minocycline, leads to a significant decrease of *P. aeruginosa* antibiotic resistance towards these antibiotics [37]. In the context of our continuing studies, we have demonstrated a stronger synergetic effect using a combination of compound **3** and a macrolide leading to an increase in Gram-negative bacteria susceptibility to this antibiotic family. Interestingly, the in vitro experiments presented different antimicrobial activity profiles depending on the considered bacterial strain, which could suggest that different hydrophobicity levels of the tested macrolides lead to different interaction pathways with the outer membrane of these pathogens. 

For further understanding of compound **3** behavior, its mechanism of action was more precisely investigated, demonstrating the strongest antimicrobial activity among all the parent derivatives tested. On the basis of the outer membrane permeabilization assay, our data suggested that compound **3** disrupts the outer membrane integrity of Gram-negative bacteria more strongly compared with polymyxin nona (used as an internal control). More interestingly, the behavior of compound **3** toward the outer membrane of the different tested strains was highly variable. This suggests that the differences encountered may be due to variation in Gram-negative bacteria outer membrane composition, implying that the role of lipid A could strongly modify the bacterial adaptation to withstand external stresses [13]. This study also demonstrated that compound **3** has a weak effect on the depolarization of the bacterial inner membrane and that this phenomenon occurs in a dose-dependent manner. Interestingly, this depolarization was twofold higher in the case of AG100A_pUC18, which does not exhibit efflux pumps. The same result was obtained by measuring ATP efflux in the presence of compound **3** where no significant differences can be noticed between the different strains, except for Ag100A_pUC18.

Taken together, these results suggest that compound **3** presents a weak effect on the inner membrane depolarization as evidenced by a weak ATP efflux level, but it strongly disrupts the integrity of the outer membrane of Gram-negative pathogens.

## 4. Methods and Materials

All the solvents were purified according to reported procedures, and the reagents used were commercially available. Methanol, ethyl acetate, and dichloromethane were purchased from Sigma-Aldrich (St. Quentin Fallavier, France) and used without further purification. Column chromatography was performed on Merck silica gel (70–230 mesh). ^1^H NMR and ^13^C NMR spectra were recorded in MeOD on a Bruker AC 300 spectrometer working at 300 MHz and 75 MHz, respectively (The usual abbreviations were used: s: singlet, d: doublet, t: triplet, q: quadruplet, and m: multiplet). All chemical shifts are given in ppm. Mass spectroscopy analysis was performed by the Spectropole Laboratory (Marseille, France). The purity of the compounds was checked by analytical HPLC (C18 column, eluent CH_3_CN-water-TFA (90:10:0.025, *v*/*v*/*v*), 0.5–1 mL/Min) with PDA detector spanning from 210 nm to 310 nm. All compounds possessed purity above 95%, as determined by analytical HPLC-PDA at 210 nm.

### 4.1. Procedure for the Synthesis of Polyaminoisoprenyl Derivatives ***3**–**6***

#### Synthesis of Compound **3**

To a solution of spermine (450 mg, 2.27 mmol) and triethylamine (450 µL, 4.5 mmol) in distilled tetrahydrofuran (THF) (10 mL) was added dropwise farnesyl chloride **1** (480 mg, 2 mmol) in THF (15 mL). The reaction mixture was stirred at room temperature for 24 h and evaporated to dryness. The crude residue was purified by column chromatography (eluant CH_2_Cl_2_/MeOH/conc. NH_4_OH, 7:3:1) to afford the pure desired compound in 64% yield as a mixture of isomers. Yellow solid; ^1^H NMR (MeOD, 300 MHz): *δ* = 5.28–4.93 (m, 3H), 2.93–2.57 (m, 14H), 2.19–1.92 (m, 10H), 1.87–1.63 (m, 23H). ^13^C (MeOD, 75 MHz): *δ* = 140.06, 139.44, 136.28, 136.25, 136.16, 136.13, 132.37, 132.13, 132.10, 125.94, 125.42, 125.38, 125.21, 123.15, 121.80, 54.58, 52.48, 52.25, 50.66, 50.52, 48.77, 48.24, 48.01, 47.63, 41.28, 41.18, 41.12, 40.94, 40.90, 40.80, 40.62, 33.26, 32.99, 30.35, 30.11, 28.53, 28.21, 27.84, 27.66, 27.43, 26.09, 26.04, 25.41, 23.84, 23.79, 17.90, 17.86, 16.61, 16.48, 16.25, and 16.21. C_25_H_50_N_4_ MS (ESI+) *m*/*z* 407.41 (100%, [M + H]^+^), and cald. 407.703.

All the other compounds **4**–**6** were synthesized according to a previous reported procedure [36] (see Figure S1).

### 4.2. Bacterial Strains

For this study, we used some of the most common Gram-negative bacteria involved in severe infections, primarily reference strains (PA01, *E. coli* ATCC 25922, *K. pneumoniae* ATCC 13883, *C. Koseri* IP8294, *E. cloacae* DSM 129, and *K. aerogenes* ATCC 13048) and a multiresistant strain *K. aerogenes* 289, as well as an *E. coli* β-lactamase-producing strain AG100A_pUC18. These strains were stored in 15% (*v*/*v*) glycerol at −80 °C for cryo-protection and sub-cultured overnight in Mueller–Hinton broth 2 (MH II) at 37 °C for inoculum preparation.

### 4.3. Transfer of Plasmid pUC-18 into Escherichia coli Strains

The different strains of *E. coli* were made competent by a calcium chloride treatment then transformed with the pUC18 plasmid by a heat shock. A single colony of AG100A was inoculated into 10 mL of Luria–Bertani (LB) medium and bacteria were grown until OD_600nm_ = 0.4 at 37 °C with shaking (180 rpm). The cultures were transferred into a centrifuge bottle, placed on ice for 20 min and centrifuged at 4000× *g* for 10 min at 4 °C. The pellets were resuspended in 5 mL of CaCl_2_ at 50 mM and incubated in ice for 1 h. Bacteria were centrifuged again, and the pellets were gently resuspended in 200 µL of CaCl_2_ 50 mM and glycerol 15%. Then, 100 ng of pUC18 was added at 100 µL of the competent strain and the cells were incubated in ice for 20 min. They were then incubated 2 min at 42 °C and transferred in ice. Subsequently, 900 µL of LB was added, and the tubes were incubated at 37 °C with shaking (180 rpm) for 1 hour. Dilutions were then prepared and spread on LB agar plates containing 100 µg/mL of ampicillin. Colonies were selected after overnight incubation at 37 °C and PCR tests were performed for verification by amplifying the gene coding for the β-lactamase on pUC18. 

### 4.4. Antibiotics

A large panel of antibiotics belonging to the macrolide family—such as erythromycin, josamycin, roxithromycin, azithromycin, spiramycin, clarithromycin, dirithromycin, and tylosin—was used in this study. All antibiotics were purchased from Sigma (St. Quentin Fallavier, France) and dissolved in water, dimethyl sulfoxide (DMSO), or ethanol as indicated. 

### 4.5. MIC Determination of Macrolides and Polyaminoisoprenyl Derivatives

The minimal inhibitory concentration (MIC) is defined as the lowest concentration of an antibiotic able to neutralize the majority (99.9%) of a bacterial inoculum. Susceptibilities to macrolides and compounds **3**–**6** were determined in sterile 96-well microplates by using the standard broth dilution method in accordance with the recommendations of the Comité de l’Antibiogramme de la Société Française de Microbiologie (CA-SFM) [43]. The stock solutions of macrolide antibiotics were freshly prepared at a 51.2 mg/mL concentration for each experiment in water, ethanol, or dimethyl sulfoxide (DMSO) as indicated. Briefly, the MICs were determined with an inoculum of 10^5^ CFU in 200 µL of MH2 broth containing twofold serial dilutions ranging from 1024 µg/mL to 2 µg/mL of each molecule. The MIC was defined as the lowest concentration of drug that completely inhibited visible growth after incubation for 18 h at 37 °C. All MIC determinations were repeated in triplicate in independent experiments. 

### 4.6. Determination of MICs of Macrolides in the Presence of Synergizing Compounds ***3**–**6***

The antimicrobial activities of macrolides in combination with compounds **3**–**6** at a 10 µM concentration were evaluated in sterile 96-well microplates. A twofold serial dilution of the drugs (from 1024 μg/mL to 2 μg/mL) was performed from the starting solution. In each column of the microplate, the concentration of the adjuvants was set at 10 μM/well. The bacterial suspension was prepared from colonies grown overnight. The concentration was adjusted to 10^5^ CFU/well. The MIC of each combination was determined after 18 h of incubation at 37 °C. All MIC determinations were repeated at least three times in independent experiments. The gain was defined as the ratio of the MIC of each macrolide antibiotic to its MIC determined in the presence of each adjuvant.

### 4.7. Chequerboard Assay/Fractional Inhibitory Concentration Index (FICI)

To determine the interaction and to evaluate the combined effect of the considered drug in the presence of compound **3**, a chequerboard method was used. This test allows for the determination of the MIC and FICI values at the same time. This test was performed in 96-well microplates. The amount of 50 µL of a twofold serial dilution of each macrolide was added in the lines. Then, 50 µL of the different concentrations of compound **3** ranging from 50 µM to 0.78 µM was dispensed in the columns. Then, 100 µL of the bacterial suspension containing 10^5^ CFU/mL was added to the different wells and the plates were incubated at 37 °C for 24 h.

The combination effects were evaluated by the sum of FICIs of the macrolide–compound **3** combination.

$$\mathrm{FICI}=\frac{\mathrm{MIC}\;\mathrm{of}\;\mathrm{macrolide}\;\mathrm{in}\;\mathrm{combination}}{\mathrm{MIC}\;\mathrm{of}\;\mathrm{macrolide}\;\mathrm{alone}}+\frac{\mathrm{MIC}\;\mathrm{of}\;\mathrm{compound}\;3\;\mathrm{in}\;\mathrm{combination}}{\mathrm{MIC}\;\mathrm{of}\;\mathrm{compound}\;3\;\;\mathrm{alone}}\;$$

Four types of effects were classified as follows: FICI ≤ 0.5 = synergistic; 0.6 < FICI ≤ 0.9 = additive; 1 < FICI ≤ 3.9 = indifferent; and FICI > 4.0 = antagonistic

### 4.8. Outer Membrane Permeabilization Assay

Nitrocefin was used as a chromogenic substrate of periplasmic β-lactamase to measure the outer membrane permeabilization. The nitrocefin hydrolysis assay is a colometric assay wherein a color change from yellow to red occurs when the chromogenic β-lactam is efficiently hydrolyzed by periplasmic β-lactamases. This test was determined on the different Gram-negative bacteria (PA01, *E. coli* ATCC 25922, *K. pneumoniae* ATCC 13883, *C. Koseri* IP8294, *E. cloacae* DSM 129, *K. aerogenes* ATCC 13048, *K. aerogenes* 289, and AG100A_pUC18) to investigate the effect of the polyaminoisoprenyl derivatives on the outer membrane.

After an overnight culture of the different bacteria at 37 °C, 100 µL of each suspension was added to 10 mL of MHII broth, except for AG100A_pUC18 whose suspension was supplemented with 100 µg/mL of ampicillin to maintain the pUC 18 plasmid. It is noteworthy that for PA01 and *E. aerogenes* ATCC 13048 suspensions, 0.001 µg/mL of imipenem was added when the cultures reached the mid-logarithmic phase (OD_600_ = 0.5) to induce the β-lactamase production. The cells were then recovered by centrifugation (3600× *g* for 20 min at 20 °C) and washed twice with 20 mM potassium phosphate buffer (pH 7.2) and 1 mM MgCl_2_ (PPB). After a second centrifugation, the pellet was resuspended and adjusted to an OD_600_ of 0.375. Then, 100 µL of each bacterial suspension was mixed with 50 µL of a solution of compounds **3**–**6** at a concentration of 128 µM already set up in a 96-well microplate. Polymyxin B (PMB) and polymyxin Nona (PMBn) were used as positive controls, and PPB was used as a negative control. Finally, 50 µL of nitrocefin was added to obtain a final concentration of 50 µg/mL. Nitrocefin hydrolysis was monitored by measuring the increase in absorbance at 490 nm using a M200 Pro Tecan spectrophotometer for 1 h with a 1-min interval between each measurement. Experiments were performed in triplicate.

### 4.9. Membrane Depolarization Assay

The different Gram-negative strains were grown in MH II broth for 24 h at 37 °C. After reaching an OD_600 nm_ of 0.5, cells were centrifuged (3600× *g* for 20 min at 20 °C) and washed twice in Hepes (5 mM) (pH = 7.2) supplemented with sucrose (250 mM final concentration) and MgCl_2_ (25 mM final concentration). The fluorescent dye 3,3′-diethylthiacarbocyanine iodide DiSC_3_(5) was added to a final concentration of 5 µM and was incubated with the suspensions for 5 min at 37 °C to allow the dye incorporation into the polarized membranes. Then, 10 µL of compound **3** (the most efficient compound) was added to 90 µL of the fluorescent suspensions at different concentrations ranging from 250 µM to 7.8 µM. Fluorescence measurements were recorded after 1 min, 5 min, 10 min, and 15 min (excitation wavelength 622 nm, emission wavelength 690 nm).

The difference in the relative fluorescence values (RFU) from the control containing only buffer and the control containing bacteria treated only with cetyltrimethylammonium bromide (CTAB 1%) was chosen as the maximum level of depolarization. Assays were performed in three independent experiments.

### 4.10. ATP Efflux Measurement

Different solutions of compound **3** were prepared in twice-distilled water with a concentration ranging from 250 to 3.91 µM. The different Gram-negative suspensions were prepared in MH II broth and were incubated at 37 °C. Then, 90 µL of each bacterial suspension was added to 10 µL of a compound **3** solution and shaken for 20 s in the incubator at 37 °C. Subsequently, 50 µL of Luceferin–Luceferase reagent (Yelen, France) was added to the mixture, and luminescent signal quantified with an Infinite M200 microplate reader (Tecan, Männedorf, Switzerland) for five seconds. ATP concentration was quantified by internal sample addition. Polymyxin B (250 µM) was used as a positive control to quantify the maximum level of ATP efflux. This assay was performed in three independent experiments.

### 4.11. Cytoxicity Assays

Cytotoxicity assessment was performed on the referenced Chinese Hamster Ovary cell line (CHO-K1, ATCC-LGC Promochem, Molsheim, France). Cells were maintained in McCoy’s 5A medium (Sigma) supplemented with 10% foetal calf serum, 1 mM glutamine, and penicillin–streptomycin (100 U·mL^−1^ and 10 μg·mL^−1^, respectively), and incubated at 37 °C in a humidified atmosphere containing 5% CO_2_. The cytotoxic effects of compounds were assessed by the colorimetric WST-1 cell proliferation assay. Briefly, a range of compounds concentrations from 30 µM to 1200 µM was incorporated in triplicate cultures, and cells were incubated at 37 °C for 24 h. At the end of the incubation period, cultures were submitted to three successive washes in phosphate buffer saline (PBS) and incubated in fresh culture medium containing 10% WST-1 for an additional 30 min. Cell viability was evaluated by the assessment of WST-1 absorbance at 450 nm in a microplate spectrophotometer MRX1 II (Dynex Technologies, Chantilly, VA, USA). The Inhibitory Concentration 50% (IC_50_) was chosen to evaluate the cytotoxicity of compounds. IC_50_ was defined as the concentration of compounds that induced a 50% decrease of viable cells.

## 5. Conclusions

An original strategy has been developed affording new polyaminoisoprenyl compounds which exhibited a strong effect on the level of macrolide antibiotics susceptibility for resistant Gram-negative bacterial strains. This activity was correlated to the level of hydrophobicity of the antibiotics as well as to the ability of the polyaminoisoprenyl derivatives to alter bacterial outer membrane integrity. For the first time, we demonstrated that weak membrane perturbation is ideal for designing an intrinsically inactive, non-toxic adjuvant for non-specific potentiation of antibiotics. Whilst further testing is required before these compounds can be approved as antibiotic adjuvants for therapeutic use, outer membrane proteins are proving to be promising targets offering hope in the ongoing battle against antimicrobial resistance. Studies are now underway to determine if this restoration of antibiotic susceptibility occurs also by a direct interaction of the molecule with the efflux pump or by another mechanism.

## Figures and Tables

**Table 1:**
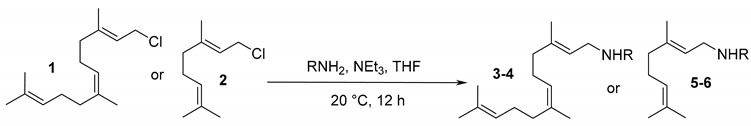
Polyaminoisoprenyl derivatives **3**–**6** synthesis from farnesyl or geranyl chloride **1**–**2**.

RNH_2_	Cpd-(Isolated Yield (%))		IC_50_ (µM) CHO	
			3–4	5–6
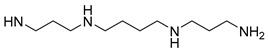	**3** (72)	**5** (64)	142.79	>150
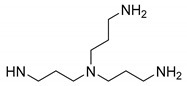	**4** (49)	**6** (63)	>150	126.82

**Table 2:** Minimum Inhibitory Concentrations of polyaminoisoprenyl derivatives **3**–**6** against various Gram-negative bacterial strains.

MIC (µM) (µg/mL)				
Strains	Cpd 3	Cpd 4	Cpd 5	Cpd 6
*P. aeruginosa* PA01	25 (10)	200 (78)	>400 (>135)	>400 (>130)
*E. coli* ATCC 25922	50 (20)	200 (78)	>200 (>67)	>200 (>65)
*K. pneumoniae* ATCC 13883	50 (20)	200 (78)	>200 (>67)	>200 (>65)
*C. koseri* IP8294	50 (20)	200 (78)	>200 (>67)	>200 (>65)
*E. cloacae* DSM 129	50 (20)	200 (78)	>200 (>67)	>200 (>65)
*K. aerogenes* ATCC 13048	100 (40)	>400 (>156)	>400 (>135)	>400 (>130)
*K. aerogenes* 289	100 (40)	>400 (>156)	>400 (>135)	>400 (>130)
AG100A_pUC18	12.5 (5)	50 (19)	100 (33)	>200 (>65)

**Table 3:** Minimum Inhibitory Concentrations of macrolides against various Gram-negative bacteria.

MIC (µg/mL)								
Strains	Erythro	Josa	Azithro	Roxithro	Spira	Clarithro	Dirithro	Tylo
PA01	512	>1024	128	>1024	>1024	512	1024	>1024
*E. coli* ATCC 25922	128	1024	8	512	512	128	64	512
*K. pneumoniae* ATCC 13883	128	1024	16	512	512	128	64	1024
*C. koseri* IP8294	256	1024	16	1024	1024	128	32	1024
*E. cloacae* DSM 129	>1024	>1024	64	>1024	>1024	1024	128	>1024
*K. aerogenes* ATCC 13048	512	512	64	512	1024	256	64	1024
*K. aerogenes* 289	>1024	>1024	64	>1024	1024	>1024	1024	>1024
AG100A_pUC18	8	32	2	32	128	64	16	256

Erythro, Erythromycin; Josa, Josamycin; Azithro, Azithromycin; Roxithro, Roxithromycin; Spira, Spiramycin; Clarithro, Clarithromycin; Dirithro, Dirithromycin; and Tylo, Tylosin.

**Table 4:** Restoration of macrolides activity (MIC values in µg/mL) against various Gram-negative bacteria in the presence of a 10 μM concentration of derivatives **3**–**6**.

Antibiotic	Cpd	PA01	E.c. ^a^	K.p. ^b^	C.k. ^c^	E.cl. ^d^	K.a. ^e^	Ea289 ^f^	E.c. ^g^
Erythromycine	**3**	128	0.5	8	2	16	4	256	<0.0005
	**4**	256	4	16	32	64	32	512	0.031
	**5**	512	64	32	64	256	128	>1024	4
	**6**	512	128	64	256	512	128	>1024	2
Josamycine	**3**	64	4	16	4	64	16	64	0.0019
	**4**	512	16	32	64	128	64	128	1
	**5**	1024	128	64	256	256	128	512	8
	**6**	>1024	512	128	512	1024	256	1024	16
Azithromycine	**3**	8	0.031	0.25	0.031	0.5	0.5	8	<0.0005
	**4**	32	1	0.5	1	4	1	16	0.25
	**5**	64	4	4	4	16	4	32	1
	**6**	64	4	4	8	32	4	64	2
Roxithromycine	**3**	512	16	16	4	64	16	512	<0.0005
	**4**	1024	16	64	32	256	64	1024	4
	**5**	1024	128	128	256	512	256	>1024	16
	**6**	1024	512	256	256	1024	256	>1024	16
Spiramycine	**3**	>1024	16	64	4	256	32	512	<0.0005
	**4**	>1024	64	128	256	512	128	256	1
	**5**	1024	256	128	512	1024	512	512	1
	**6**	>1024	256	256	1024	1024	1024	512	1
clarithromycine	**3**	32	4	4	2	8	1	512	0.0039
	**4**	128	8	8	4	64	8	1024	16
	**5**	128	16	32	32	256	64	1024	16
	**6**	128	32	32	64	512	64	1024	32
Dirithromycine	**3**	64	8	8	4	16	4	128	<0.0005
	**4**	512	16	16	8	64	8	256	2
	**5**	512	16	16	16	64	32	512	4
	**6**	512	32	16	16	128	32	512	8
Tylosine	**3**	512	16	128	4	128	32	128	4
	**4**	>1024	256	256	128	1024	256	256	32
	**5**	>1024	512	512	512	>1024	512	512	64
	**6**	>1024	512	512	512	>1024	512	1024	128

^a^ *E. coli* ATCC 25922. ^b^ *K. pneumoniae* ATCC 13883. ^c^ *C. koseri* IP8294. ^d^ *E. cloacae* DSM 129. ^e^ *K. aerogenes* ATCC 13048. ^f^ *K. aerogenes* 289 (MDR). ^g^ *E. coli* AG100A_pUC18.

**Table 5:** Fractional Inhibitory Concentration Index (FICI) obtained for interactions of compound **3** with eight different macrolides against various Gram-negative bacteria and classified as follows: FICI ≤ 0.5 = synergistic (yellow); 0.6 < FICI ≤ 0.9 = additive (green); and 1 < FICI ≤ 3.9 = indifferent (red). The variation of the color from yellow to green is related to the importance of the synergy (from higher to lower synergy).

Strain	FICI							
	Erythro	Josa	Azithro	Roxithro	Spira	Clarithro	Dirithro	Tylo
Pa01	0.65	0.46	0.46	0.90	1.40	0.46	0.46	0.90
E.c. ^a^	0.20	0.20	0.20	0.20	0.23	0.23	0.33	0.23
K.p. ^b^	0.26	0.22	0.22	0.23	0.33	0.23	0.33	0.33
C.k. ^c^	0.21	0.20	0.20	0.22	0.20	0.22	0.33	0.20
E.cl. ^d^	0.22	0.26	0.21	0.26	0.45	0.21	0.33	0.33
K.a. ^e^	0.11	0.13	0.11	0.13	0.13	0.10	0.16	0.13
Ea289 ^f^	0.35	0.16	0.23	0.60	0.60	0.60	0.23	0.23

^a^ *E. coli* ATCC 25922. ^b^ *K. pneumoniae* ATCC 13883. ^c^ *C. koseri* IP8294. ^d^ *E. cloacae* DSM 129. ^e^ *K. aerogenes* ATCC 13048. ^f^ *K. aerogenes* 289 (MDR).

## Data Availability

Not applicable.

## Supplementary Materials

The following supporting information can be downloaded at: https://www.mdpi.com/article/10.3390/ijms232012457/s1.
