# Supplementary material for: An Original and Efficient Antibiotic Adjuvant Strategy to Enhance the Activity of Macrolide Antibiotics against Gram-Negative Resistant Strains
Source: Int J Mol Sci. 2022 Oct 18;23(20):12457. doi: 10.3390/ijms232012457 (PMC9604434; doi:10.3390/ijms232012457)
Supplement: Supplementary file 1 [file ijms-23-12457-s001.zip › ijms-1932881-supplementary.pdf]

## Supporting Informations

# An original and efficient antibiotic adjuvant strategy to enhance the activity of macrolide antibiotics against Gram-negative resistant strains

Azza Troudi, Jean Michel Bolla, Naouel Klibi and Jean Michel Brunel

**General procedure for the synthesis of polyaminoisoprenyl derivatives 3**

To a solution of spermine (450 mg, 2.27 mmol) and triethylamine (450 µL, 4.5 mmol) in distillated tetrahydrofurane (THF) (10 mL) was added dropwise farnesyl chloride **1** (mixture of isomers) (480 mg, 2 mmol) in distillated THF (15 mL). The reaction mixture was stirred at room temperature for 24 h. and evaporated to dryness. The crude residue was purified by column chromatography (eluant $CH_2Cl_2$/MeOH/conc. $NH_4OH$, 7:3:1) to afford the pure desired compound as a yellow solid in 64% yield ( mixture of isomers).

**Compound 3**. 64% yield; Yellow solid; $^1$H NMR (MeOD, 250 MHz): $\delta$ = 5.22-5.25 (m, 1H), 5.03-5.14 (m, 2H), 3.11-3.25 (m, 3H), 2.50-2.73 (m, 9H), 2.43-2.49 (m, 3H), 1.96-2.11 (m, 10H), 1.69-1.74 (m, 12H), 1.50-1.67 (m, 10H). $^{13}$C (MeOD): $\delta$ = 140.06, 139.44, 136.28, 136.25, 136.16, 136.13, 132.37, 132.13, 132.10, 125.94, 125.42, 125.38, 125.21, 123.15, 121.80, 54.58, 52.48, 52.25, 50.66, 50.52, 48.77, 48.24, 48.01, 47.63, 41.28, 41.18, 41.12, 40.94, 40.90, 40.80, 40.62, 33.26, 32.99, 30.35, 30.11, 28.53, 28.21, 27.84, 27.66, 27.43, 26.09, 26.04, 25.41, 23.84, 23.79, 17.90, 17.86, 16.61, 16.48, 16.25, 16.21. $C_{25}H_{50}N_4$ MS (ESI+) m/z 407.41 (100%, $[M + H]^+$).

# $^{1}H$ and $^{13}C$ NMR Spectra of 3

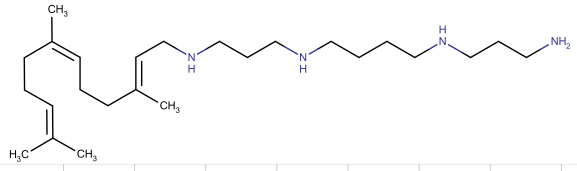


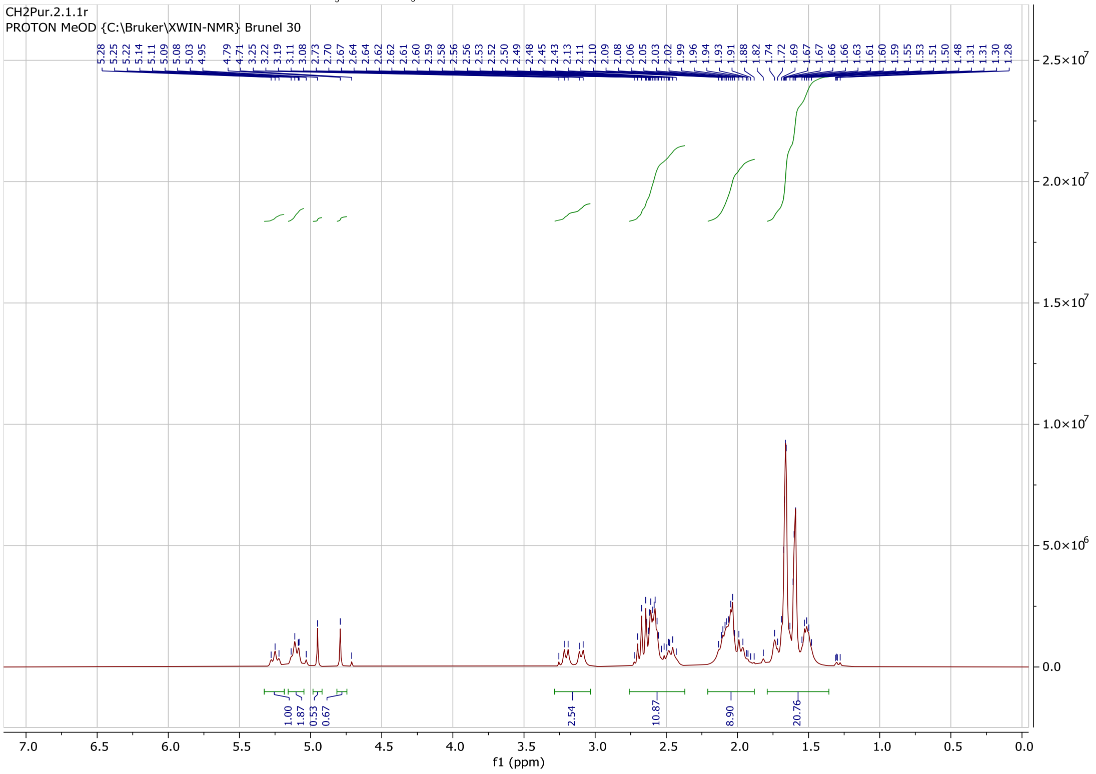


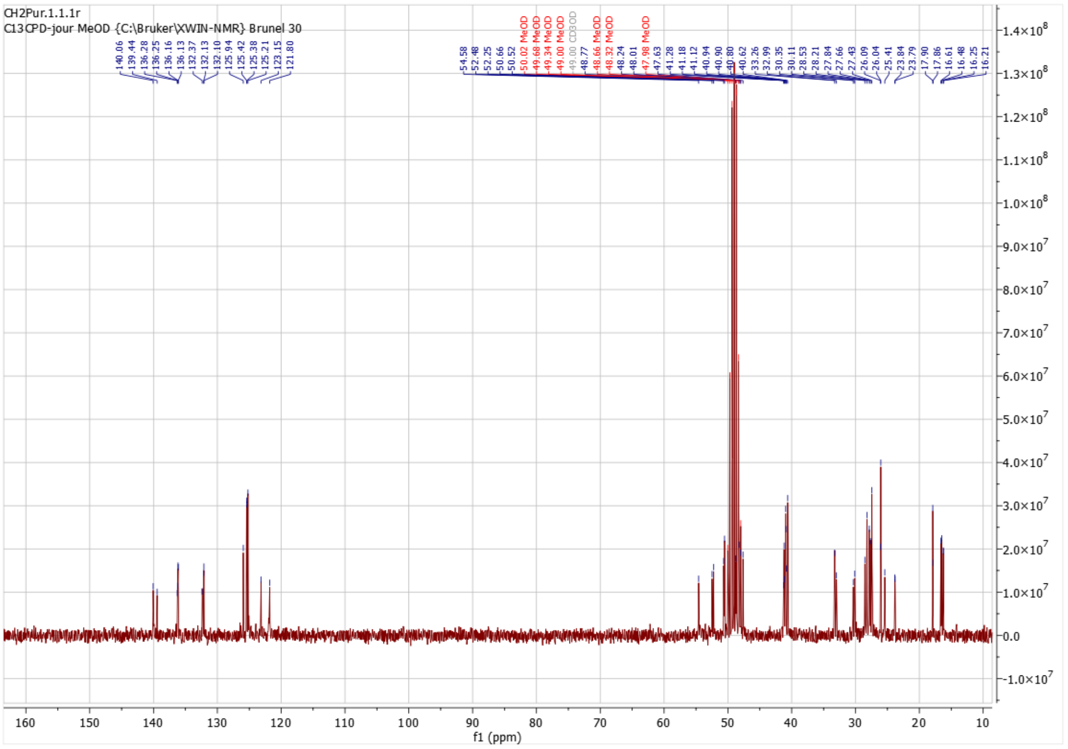


# $^{1}H$ and $^{13}C$ NMR Spectra of 4

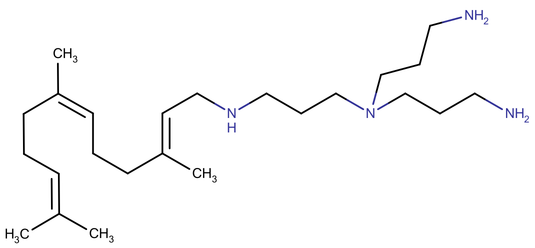


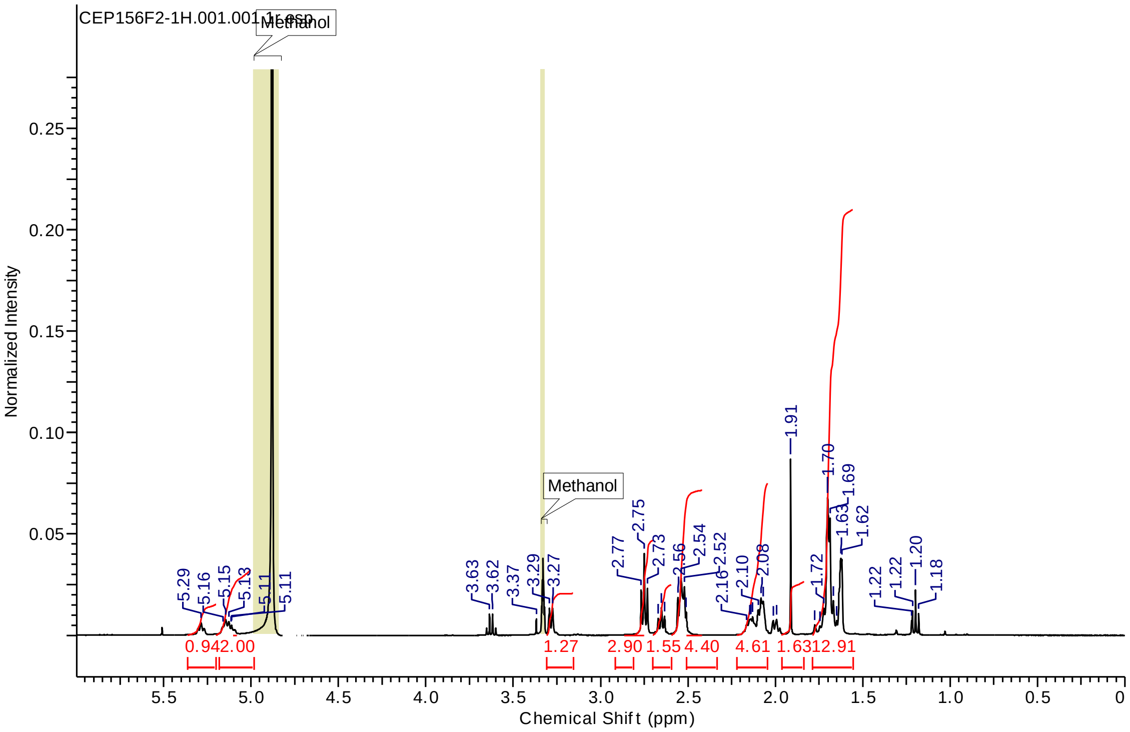


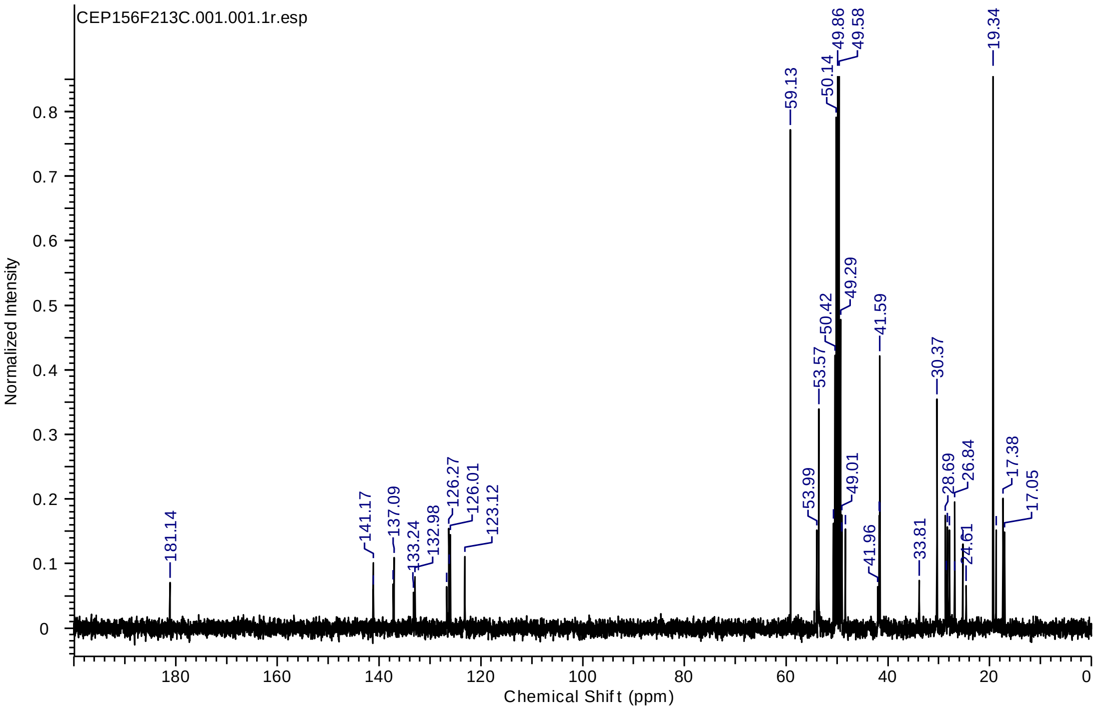


**$^{1}$H and $^{13}$C NMR Spectra of 5**

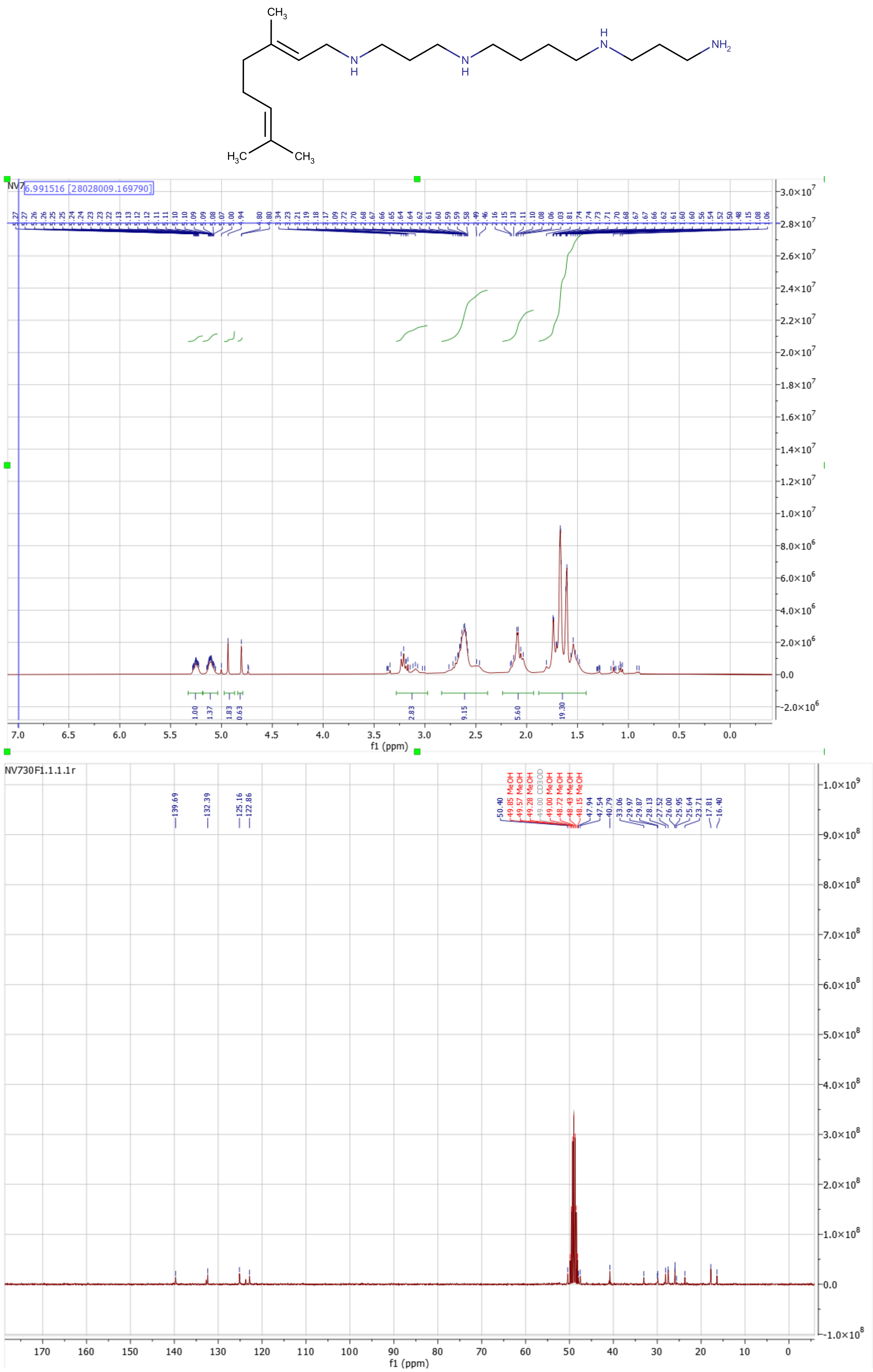


**$^{1}$H and [13]C Spectra of 6**

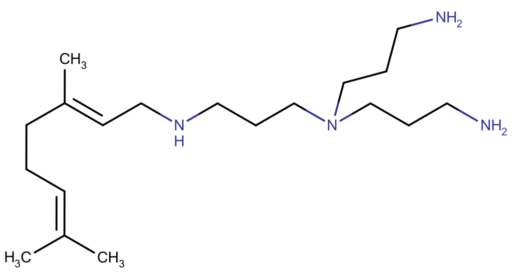


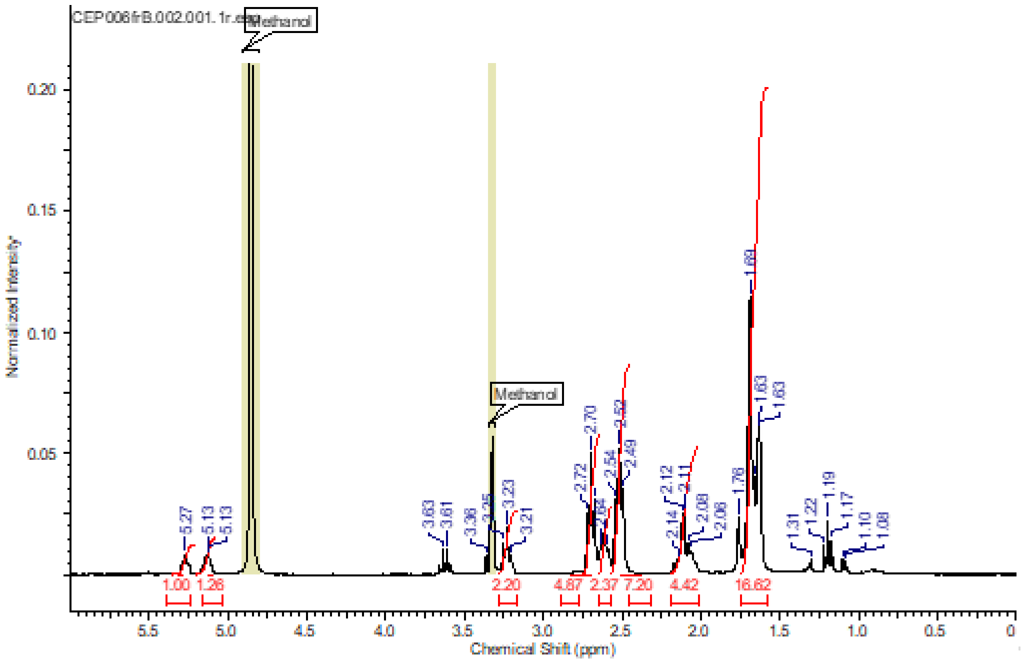


C NMR spectra of derivatives 3-6

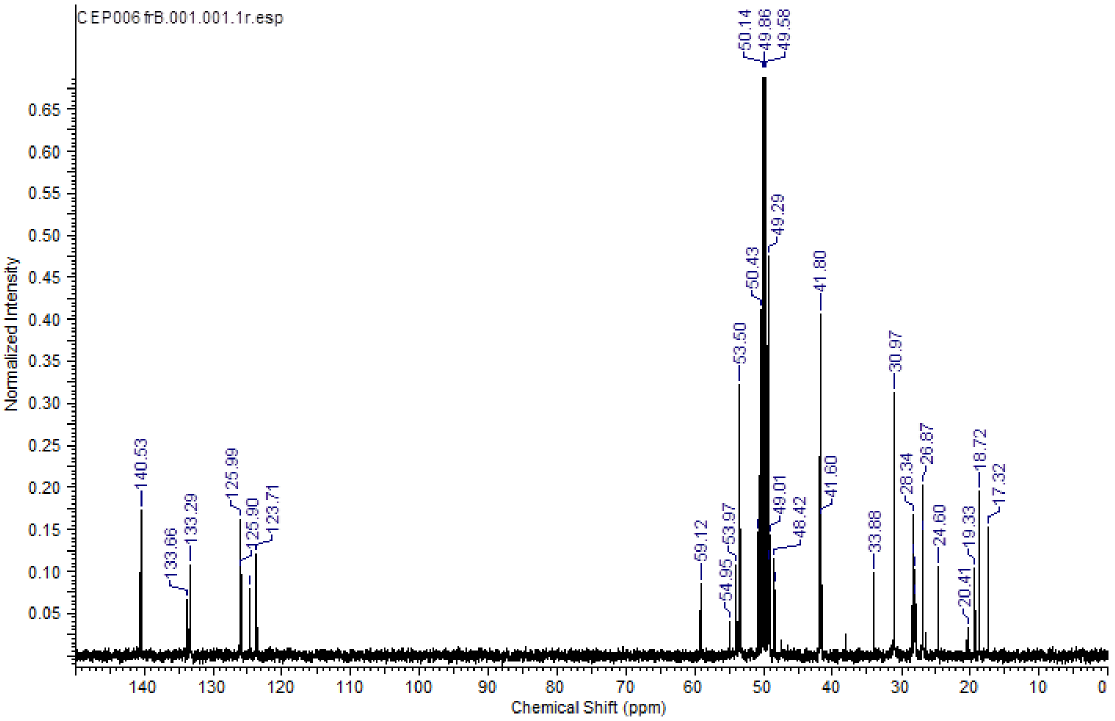


**Figure S1.** $^{1}$H and $^{13}$C NMR spectra of derivatives **3-6**

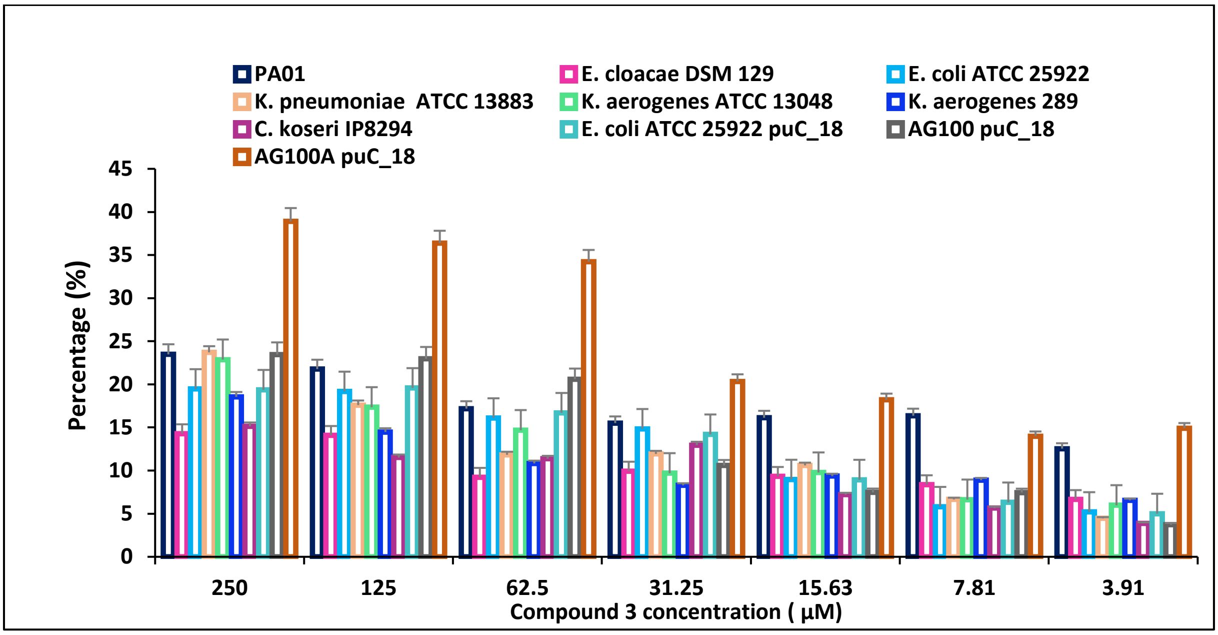


**Figure S2.** Dose-dependent inner membrane depolarization of various Gram-negative bacteria in the presence of compound **3**

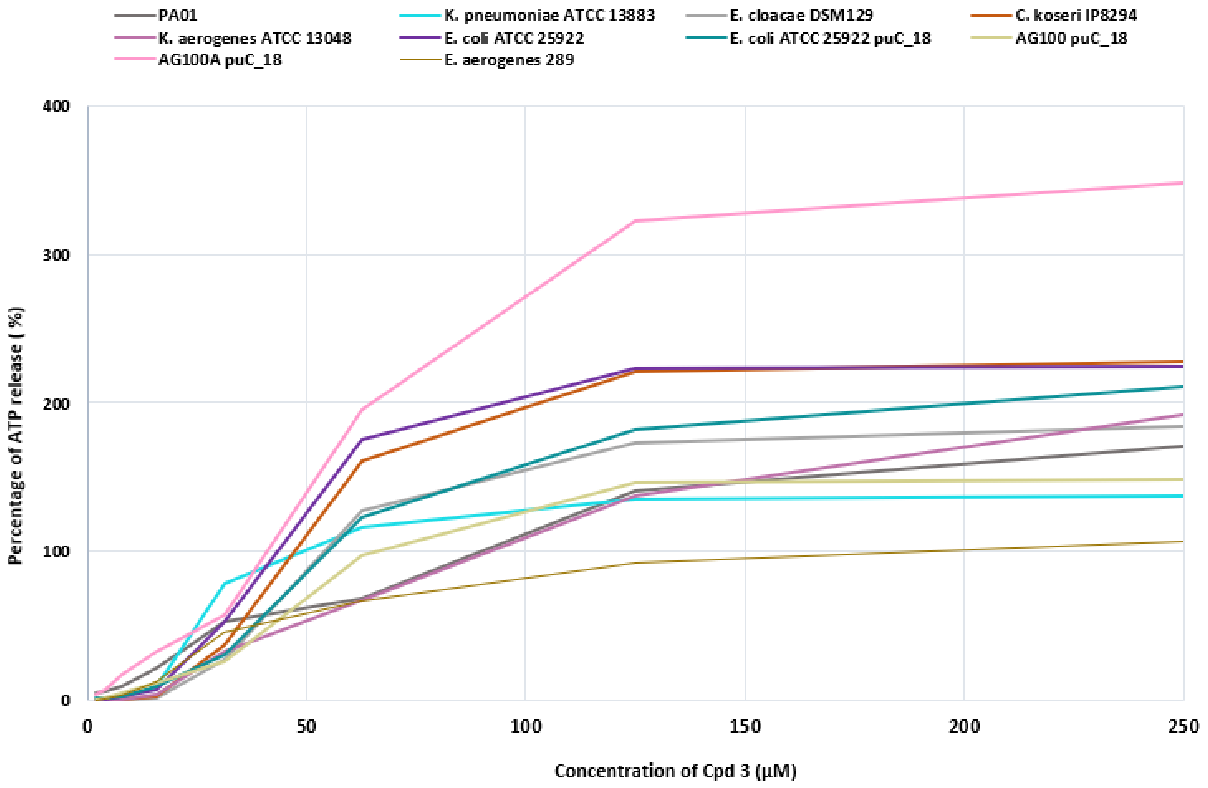


**Figure S3.** Dose-dependent ATP release levels of various Gram-negative bacteria evaluated by bioluminescence in the presence of compound **3**

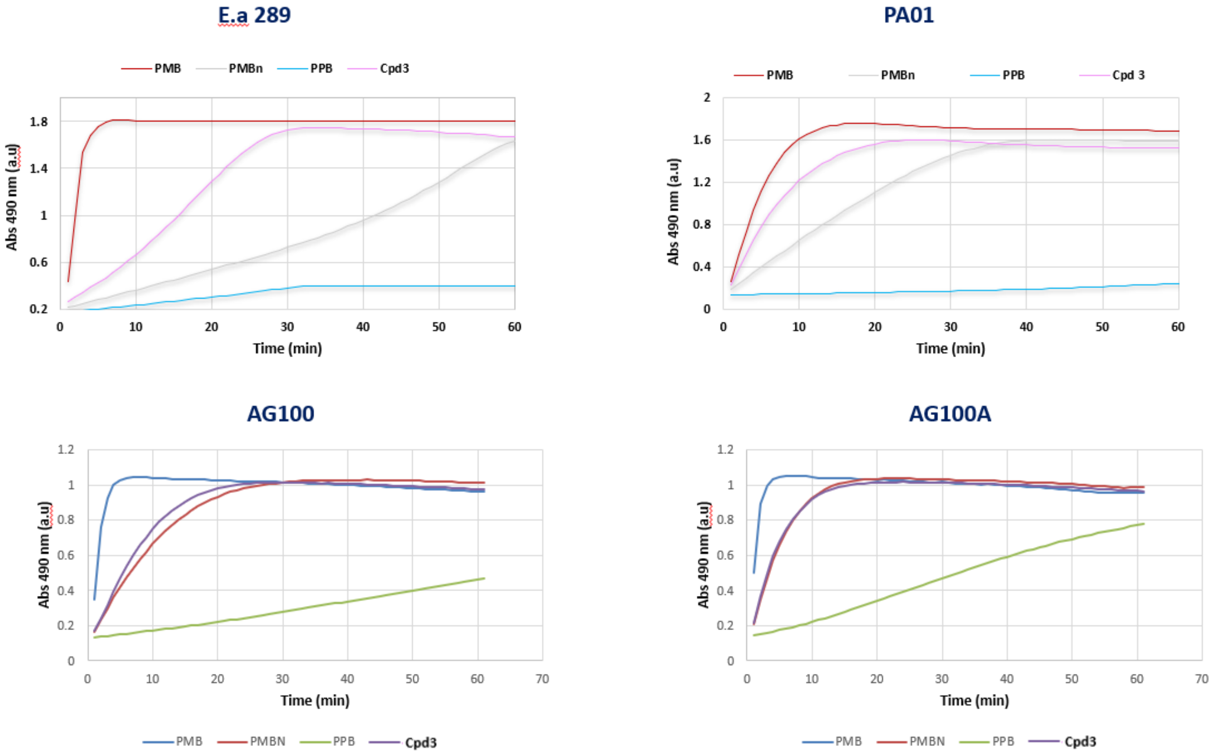


**Figure S4.** Comparison of the nitrocefin hydrolysis kinetics in the presence of PMB, PMBn, **3** used at a 125 µM concentration in the case Ea289, PA01, *E. coli* AG100, *E. coli* AG100A bacterial strains
